# Microsecond time-resolved X-ray diffraction for the investigation of fatigue behavior during ultrasonic fatigue loading

**DOI:** 10.1107/S1600577519008518

**Published:** 2019-08-20

**Authors:** T. Ors, N. Ranc, M. Pelerin, V. Michel, V. Favier, O. Castelnau, C. Mocuta, D. Thiaudière

**Affiliations:** a Laboratoire PIMM, CNRS, ENSAM, HESAM, 151 Boulevard de l’Hôpital, 75013 Paris, France; b Synchrotron SOLEIL, L’Orme des Merisiers, Saint-Aubin, BP 48, 91192 Gif-sur-Yvette, France

**Keywords:** ultrasonic fatigue tests, very high cycle fatigue, time-resolved stress measurement, laser extensometry, X-ray diffraction

## Abstract

A new method based on time-resolved X-ray diffraction is proposed in order to measure the elastic strain and stress during ultrasonic fatigue loading experiments. Pure Cu was chosen as an example material for the experiments using a 20 kHz ultrasonic fatigue machine mounted on the six-circle diffractometer available at the DiffAbs beamline on the SOLEIL synchrotron facility in France. A two-dimensional hybrid pixel X-ray detector (XPAD3.2) was triggered by the strain gage signal in a synchronous data acquisition scheme (pump–probe-like). The method enables studying loading cycles with a period of 50 µs, achieving a temporal resolution of 1 µs. This allows a precise reconstruction of the diffraction patterns during the loading cycles. From the diffraction patterns, the position of the peaks, their shifts and their respective broadening can be deduced. The diffraction peak shift allows the elastic lattice strain to be estimated with a resolution of ∼10^−5^. Stress is calculated by the self-consistent scale-transition model through which the elastic response of the material is estimated. The amplitudes of the cyclic stresses range from 40 to 120 MPa and vary linearly with respect to the displacement applied by the ultrasonic machine. Moreover, the experimental results highlight an increase of the diffraction peak broadening with the number of applied cycles.

## Abstract 2

The time-resolved X-ray diffraction technique is proposed to characterize the fatigue behavior of metallic materials during ultrasonic cyclic loading.

## 1. Introduction   

Many mechanical structures are submitted to repeated loadings during their service and can break under stresses lower than their ultimate tensile stress, especially if deformation is repeated for a very large number of cycles. This phenomenon, called the fatigue of materials, can be encountered in many industrial sectors such as transport and energy. Fatigue design is thus a crucial step in mechanical engineering and it requires a precise characterization of material behavior under repeated loadings to ensure the safety and reliability of the structures throughout their life. It is presently common to find mechanical systems subjected to several billion fatigue cycles, in what is called the gigacycle fatigue or very high cycle fatigue (VHCF) domain (Bathias & Paris, 2005 ▸).

The characterization of the fatigue behavior of materials requires fatigue tests, during which the specimen is loaded cyclically, to be conducted at different stress amplitudes until fracture. Using standard laboratory fatigue rigs operating at a few tens of Hz, one test typically needs several months or even more to reach the onset of the VHCF domain. To reduce the testing time, new approaches using ultrasonic fatigue machines have been developed during the last decades, which are based on a severe increase of the loading frequency in order to characterize the fatigue behavior within a few hours only. Ultrasonic fatigue machines typically operate at 20 kHz and allow generating so-called S/N curves (stress amplitude versus the number of cycles at failure) in the VHCF domain.

In parallel, other methods based on interrupted tests at various stress amplitudes with complementary measurement like self heating have been developed for a fast determination of the fatigue properties of the materials (Luong, 1995 ▸; Munier *et al.*, 2014 ▸). Many authors make use of this technique to predict the fatigue limit, but the method does not work for all materials because the relationship between fatigue damage and self heating remains complex. An alternative is to estimate the amount of energy stored or released by the specimen during its deformation, which is related to the evolution of crystal lattice defects and internal stresses. This stored energy is a better signature of the fatigue damage and can be estimated from the intrinsic dissipation and the mechanical work supplied to the specimen. The quantification of the last quantity necessitates the measurement of the evolution of the stress and the total strain during one fatigue cycle (Chrysochoos *et al.*, 2008 ▸; Connesson *et al.*, 2011 ▸; Mareau *et al.*, 2013 ▸).

The purpose of this paper is to present a time-resolved X-ray diffraction (XRD) approach which enables the evolution of the mechanical state of the material to be followed during a single cycle and throughout high-frequency fatigue tests.

X-ray diffraction provides information about the mean value of the elastic strain and the distribution of elastic strain in the material, at the scale of the diffracting volume. The mean elastic strain in that volume, deduced from the shift of diffraction peak positions, allows the macroscopic applied stress to be estimated thanks to a scale transition model, whereas the fluctuation of elastic strain, deduced from peak broadening, provides information about intragranular strain heterogeneities and dislocation density (Bretheau & Castelnau, 2006 ▸).

The main challenge of this kind of measurement is the temporal resolution. At 20 kHz, the duration of one cycle is 50 µs and therefore a temporal resolution in the microsecond range is necessary to correctly describe a single cycle. Time-resolved XRD measurements have been developed and used for several decades in different areas of physics, chemistry, biology and materials science. The order of magnitude of the time resolution of these techniques lies between milliseconds and femtoseconds. The domain between femtosecond and nanosecond is mainly of interest to the solid-state physicists (Wark, 1996 ▸), particularly through the study of short-term crystal structure changes (Robinson *et al.*, 2016 ▸; Fons *et al.*, 2014 ▸), formation of crystalline structures during chemical reactions, effect of high pressures related to the propagation of a shock wave (Luo *et al.*, 2012 ▸), rotation of side chains of proteins, *etc*. The domain between the microsecond and the millisecond opens the field to numerous other applications in engineering and material science (Gorfman, 2014 ▸): crack propagation (Rack *et al.*, 2014 ▸, 2016 ▸), fatigue of materials (Park *et al.*, 2007 ▸), piezo electric response of materials (Cornelius *et al.*, 2017 ▸), stress measurement in a rotating engine (Baimpas *et al.*, 2013 ▸) or in the outer raceway of a bearing test (Mostafavi *et al.*, 2017 ▸), *etc*.

The principle of time-resolved XRD is based on pump–probe methods. The material is loaded by external stress (the pump) which may be periodic: electric field, temperature field, mechanical stress, laser pulse. The probe is composed of a continuous or pulsed X-ray source and a detector, this last one allowing extremely short exposure times and signal accumulation over several identical cycles. The measurement is carried out by controlling the delay between the pump and the probe. There are two ways of performing time-resolved XRD with pump–probe methods: for the first one, which corresponds to time resolutions under 100 ns, the X-ray source is pulsed (*e.g.* synchrotron radiation in pulse mode or X-ray free-electron laser), and the time resolution of the measurement is directly associated with the pulse duration. This technique allows very high resolutions to be reached, of the order of femtoseconds. In the second method, where the time resolution is higher than 100 ns, the X-ray source is considered as continuous (which can be pulsed, but with a frequency much higher than the data acquisition one), and the time resolution is directly related to the counting and the data transfer time of the detector. If the phenomenon to be observed can be repeated cyclically, it is possible to use stroboscopic methods to either reconstruct a temporal evolution by changing the delays between the pump and the probe (especially for very short times) or simply to increase the number of cycles over which data are accumulated, *i.e.* total integration times of the signal to obtain diffraction patterns with better counting statistics. In the case of ultrasonic fatigue with the conditions detailed here, the time resolution needs to be about 1 µs, and the latter method mentioned above with an X-ray beam that is assumed to be continuous will be used, as detailed below. Taking into account the low stress levels in the VHCF domain, the fatigue damage can be considered constant over approximately 10^5^ cycles and it will therefore be possible to apply the stroboscopic method.

The main objective of this paper is to present the development and the implementation of a pump–probe method during ultrasonic fatigue tests with the stroboscopic method in order to reconstruct loading cycles with the help of diffraction patterns. After this introduction, the second section of this paper will be devoted to the experimental methodology. In the third section, the diffraction pattern correction and analysis (bad pixel detection, geometrical corrections and diffraction peak fitting) will be detailed. Finally, in the last section, the experimental results will be presented and discussed.

## 2. Experimental methodology   

### 2.1. Experimental setup   

**Figure 1:**
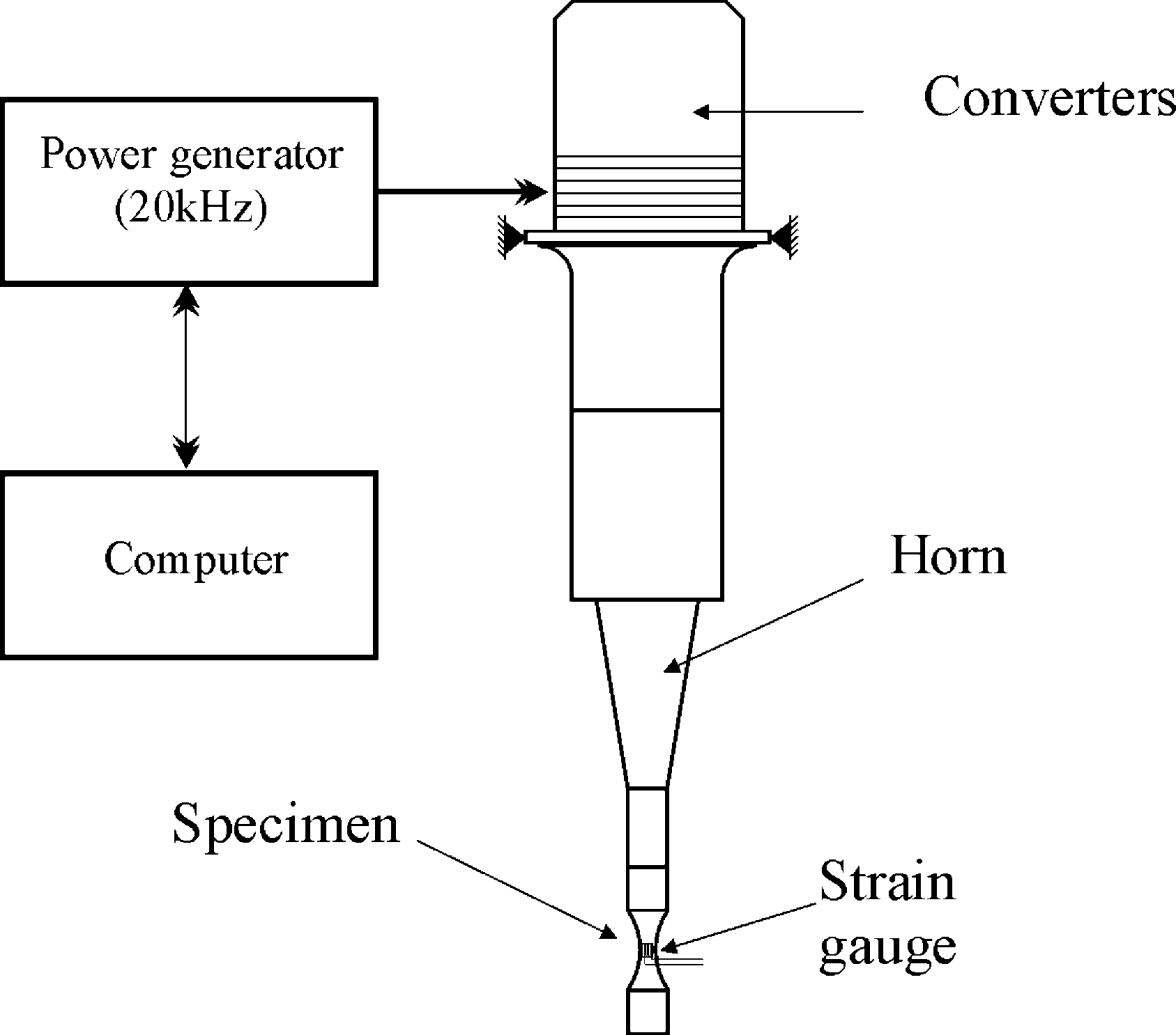
Schematic view of the used ultrasonic fatigue machine. The detailed sample geometry is shown in Fig. 2 ▸.

**Figure 2:**
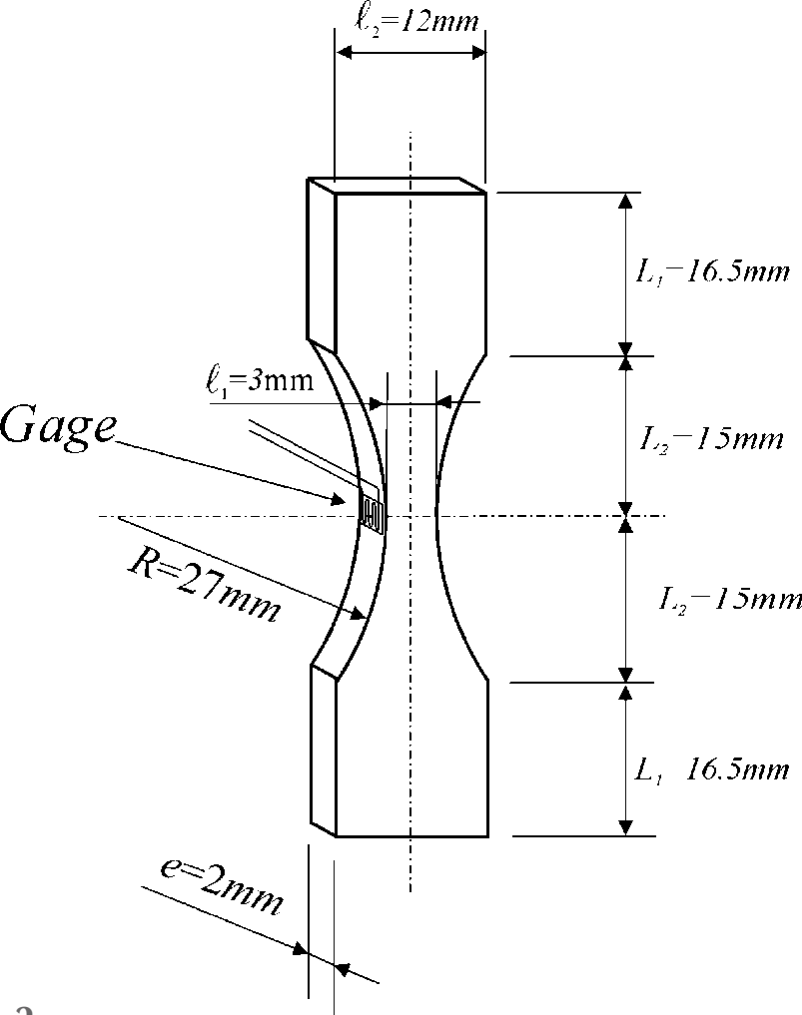
Geometry of the ultrasonic fatigue sample.

The fatigue loading is applied by an ultrasonic fatigue machine. The technology of this ultrasonic machine is completely different from that of conventional fatigue machines and is based on the vibration of a free-standing specimen in its first longitudinal mode. The specimen vibration is induced by a piezoelectric converter and a horn that amplifies the vibration amplitudes (Fig 1 ▸). The machine is controlled via the tension applied to the piezoelectric converter and therefore the vibration amplitude is imposed on one edge of the specimen. The relationship between the piezoelectric tension amplitude and the displacement on the upper edge of the specimen is determined by calibration. The stress distribution along the specimen and the maximum stress are obtained using a harmonic calculation and assuming a linear elastic behavior of the material constituting the specimen. The used elastic hypothesis is not completely correct because fatigue loading is always accompanied by irreversible deformation mechanisms which explain fatigue failure. However, deviations from ideal elastic behavior may be negligible for small stress amplitudes. Moreover, the total strain (*i.e.* elastic + plastic strains) in the center of the specimen is directly measured by two thermally compensated strain gages glued on the right and left sides of the bone-shaped specimen (Fig. 2 ▸) and mounted in a Wheatstone half-bridge. The output gage signal is conditioned and amplified with a high cut-off frequency of at least 500 kHz.

Different specimen materials were tested to develop and verify the method we introduce in this paper: pure and alloyed aluminium, dual phase steel and pure copper. Throughout this paper, results on pure copper will be presented as an illustrative example. Pure copper sheets produced by cold rolling were cut into final sample dimensions as shown in Fig. 2 ▸. The samples were polished mechanically, then electrolytically to remove surface hardening produced by sample machining that may lead to peak broadening and peak shape change. Careful sample polishing also helps eliminating surface residual stresses that may influence fatigue behavior. The grain size was determined to be about 10–20 µm by electron microscopy methods. Two independent Bragg reflections of this face-centred cubic structure were measured: 311 and 220, corresponding to Bragg angles 2θ_theo_ of 41.65° and 35.30°, respectively, for an incoming monochromatic X-ray beam of 16 keV.

To carry out X-ray diffraction, the ultrasonic machine was installed on the six-circle diffractometer of the DiffAbs beamline of the SOLEIL synchrotron.

**Figure 3:**
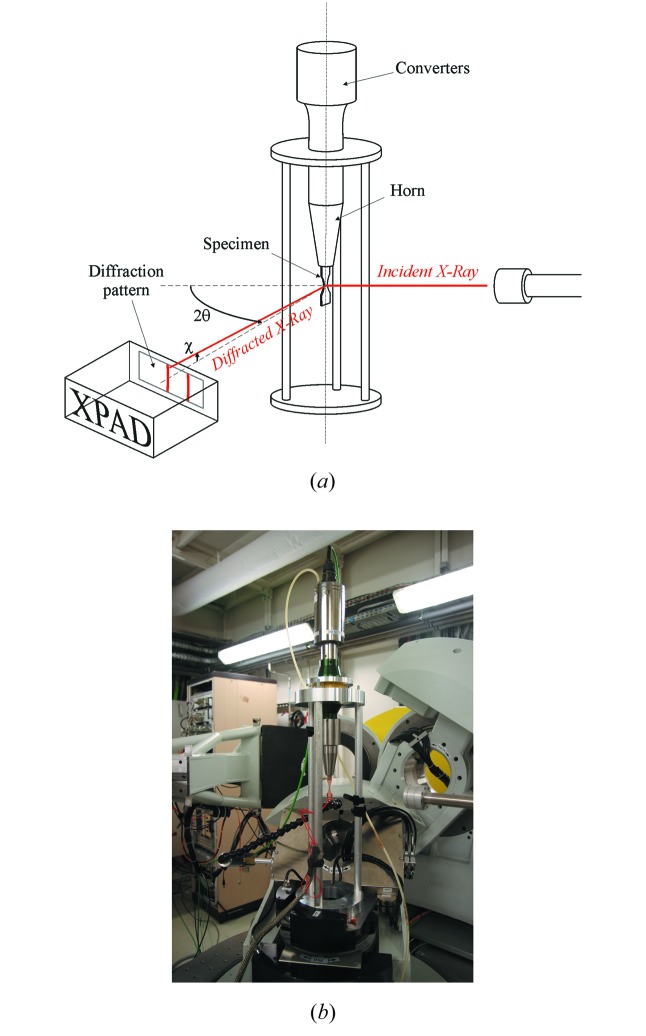
(*a*) Schematic of the configuration used for the experiment and (*b*) photograph of the ultrasonic machine installed on the diffractometer on the DiffAbs beamline.

The incoming beam size [full width at half-maximum (FWHM), horizontal × vertical] was about 290 µm × 220 µm. The diffraction patterns were acquired with a 2D hybrid pixel X-ray detector [XPAD3.2, pixel size = 130 µm × 130 µm, 960 × 560 pixels (Le Bourlot *et al.*, 2012 ▸; Medjoubi *et al.*, 2010 ▸)]. One of the most interesting features of this detector is the availability of an externally triggered electronic shutter that allows photons to be counted within a time interval as small as ∼100 ns and with the possibility of accumulating the X-ray scattered signal for a defined number of triggers. This allows to precisely synchronize the very short detector time aperture (1 µs in our case) with the gage signal recording the total specimen strain, all the while the specimen is deformed under fatigue. The detector was placed 630 mm away from the sample surface, with its long dimension along the horizontal direction [see Fig. 3(*a*) ▸] as our actual ultrasonic fatigue rig requires holding the specimen in the vertical direction. A schematic view of the setup as well as a photograph of the ultrasonic machine installed on the goniometer is shown in Fig. 3 ▸. The experiment was conducted in reflection geometry, with a fixed incidence angle (19.3°) between the X-ray beam and the sample surface corresponding approximately to the midpoint of the 311 and 220 reflections. Therefore, diffraction vectors for the mentioned peaks are slightly misaligned with respect to each other and not exactly perpendicular (but close to) to the specimen surface.

### 2.2. Triggering of the detector and XRD data collection   

**Figure 4:**
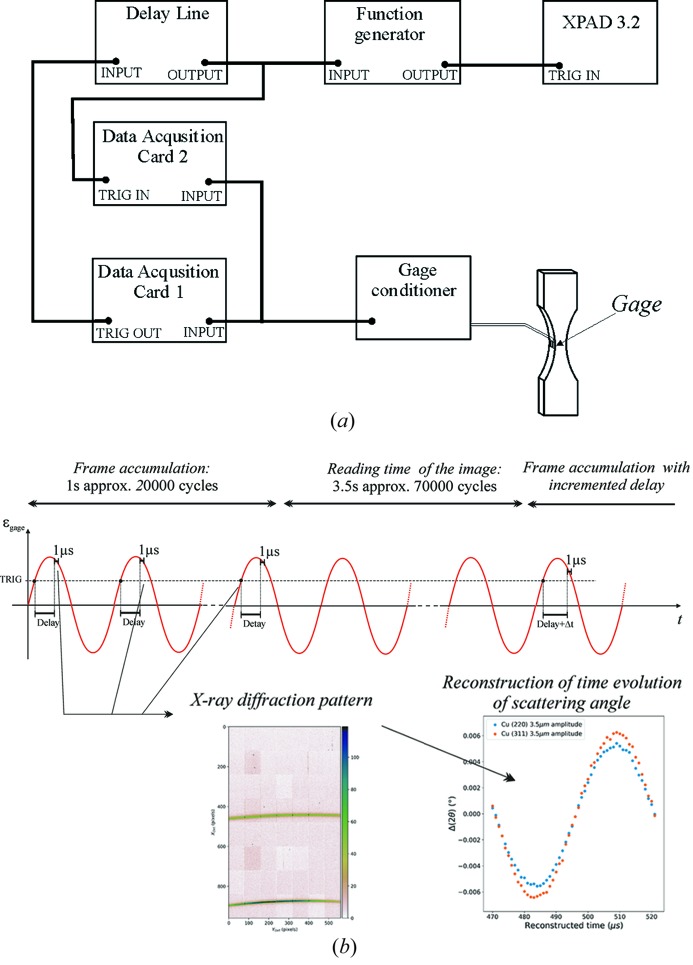
(*a*) Schematic view of the electronics chain used for data acquisition. (*b*) Principle of the triggering method.

As mentioned in the *Introduction* [Sec sec1], in the case of ultrasonic cyclic loading, a stroboscopic method is used to reconstruct the temporal evolution of the specimen during one cycle. Therefore, a triggering procedure for the detector was used, as described in Fig. 4 ▸. The ‘zero delay’ trigger signal [labeled as TRIG in Fig. 4(*b*) ▸] is set when the gage signal reaches a given value (0.5 V in our experiments). This corresponds to a given deformation level (or stress value) within the cyclic fatigue loading. After a certain adjustable time delay, the system sends a trigger signal to the XPAD detector to start acquisition for a short time (here 1 µs). A schematic view of the electronic chain which assures the triggering of the XPAD detector is shown in Fig. 4(*a*) ▸. It is composed of a first data acquisition card which acquires the gage signal and gives a ‘zero delay’ TTL signal (Transistor–Transistor Logic signal) to the input of the delay line device. After a given delay, the delay time device triggers (i) the acquisition of the actual gage signal during 1 µs using the second acquisition card and (ii) the data acquisition and the time aperture of the XPAD detector for the same duration. Before arriving at the detector, the signal goes through a function generator. This step handles the re-shaping of the input from the delay line to have the right shape (a pulse wave) with a pulse width that defines the aperture time (1 µs in this case). This triggering process is repeated exactly the same way during many cycles (*i.e.* each 50 µs), until the accumulated intensity on the XPAD image is sufficient to achieve good counting statistics in order to reduce the relative uncertainties of the measured intensities [Fig. 4(*b*) ▸]. Then, the image is read and stored. For the next image, the time delay is slightly increased (here by Δ*t* = 1 µs step) and the process is repeated until a complete cycle is described, *i.e.* 50 images acquired [Fig. 4(*b*) ▸]. Then the results from each image (*i.e.* diffraction peak position) are extracted and reported as a function of the delay time in order to reconstruct a full cycle [Fig. 4(*b*) ▸]. A drawback of this procedure is that only 2% of the photons are used, as one has to wait for 49 µs between two openings of the electronic shutter.

To define the number of cycles during which photons should be accumulated in order to measure the elastic strain with an accuracy required by this mechanical study, different tests have been carried out, with various counting durations. The procedure is detailed in Appendix *A* [App appa]. It has been found that a strain resolution of 6 × 10^−6^ can be reached when the detector is triggered 20000 times for each cumulated XPAD image. Doing so, an accumulation during 20000 cycles lasts for 1 s although the detector captures photons during 20 ms. Taking into account the reading time, the time to obtain one diffraction image is about 4.5 s. Fifty such images are needed to reconstruct the shape of a single cycle and the time required to register them corresponds to 4.5 × 10^6^ cycles performed by the ultrasonic machine, which is still a small number compared with the number of cycles at fracture in the gigacycle fatigue domain (VHCF). Thus, it is supposed that during this acquisition cycle the fatigue properties of the material does not change.

## 3. Analysis of diffraction data   

### 3.1. Correction of the intensity data   

Since we are interested in very high cycle fatigue, the stress applied to the sample is relatively low, usually between 10 and 100 MPa. Accordingly, the setup and data treatment should allow a resolution to be reached for the relative strain fluctuation of the order of 10^−5^ (or stress fluctuation of ∼1 MPa), *i.e.* capturing peak shifts as small as ∼0.001°. Measuring at such an angular resolution requires additional correction steps taking the device geometry and detector response into account.

**Figure 5:**
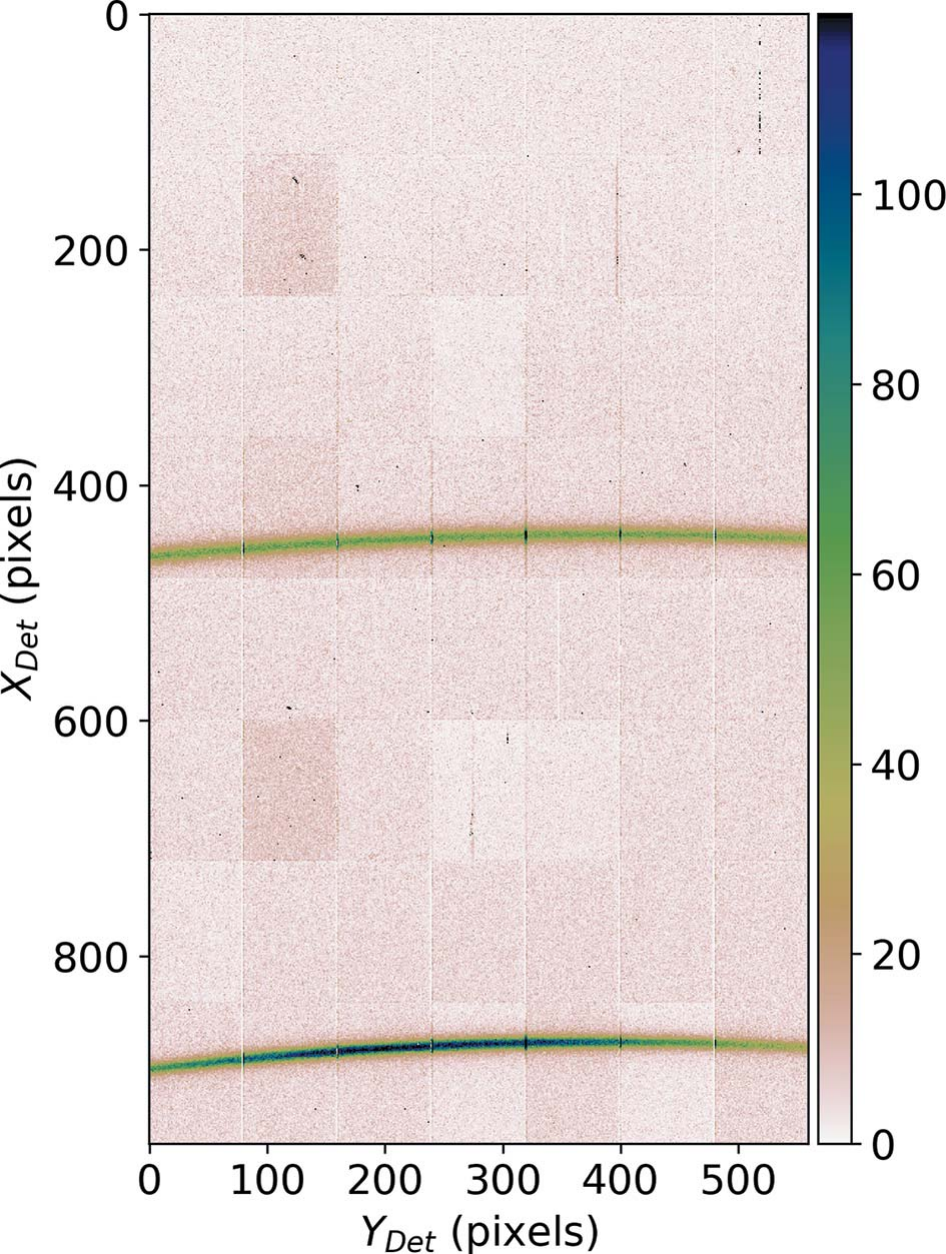
The raw 2D diffraction pattern of 960 × 560 pixels recorded by XPAD3.2.

The used XPAD3.2 detector consists of eight modules, each of them being composed of seven chips (80 × 120 pixels on each chip). The modules are stacked to form the whole detector surface, but are assembled slightly tilted and with a gap of about 3.5 mm in between each of them. A more precise description of the active area of this detector can be found by Mocuta *et al.* (2013 ▸). Consequently the diffraction pattern is read as a 2D image of 960 × 560 pixels (see Fig. 5 ▸).

The detector response (conversion of incoming photons to counts) contains a few irregularities as is the case for all 1D and 2D detectors. Firstly a line of ‘double’ pixels (their exact size is in fact 325 µm × 130 µm) exists at the junctions between each of the chips. The intensity data observed for such pixels are not necessarily reliable as photons have a different chance of hitting them and they cover a different angular range than the regular pixels. The data obtained from double pixels therefore must be corrected or rejected. In addition to this, hot, cold and dead pixels exist. Hot pixels are those which have a high photon count regardless of the measurement parameters. Cold pixels are those which yield counts systematically lower than a regular pixel and the dead pixels stay around zero level at all times. The number of these pixels (hot, cold and dead) is not static and can evolve through time with usage. One needs to identify all such pixels and their data must be rejected.

**Figure 6:**
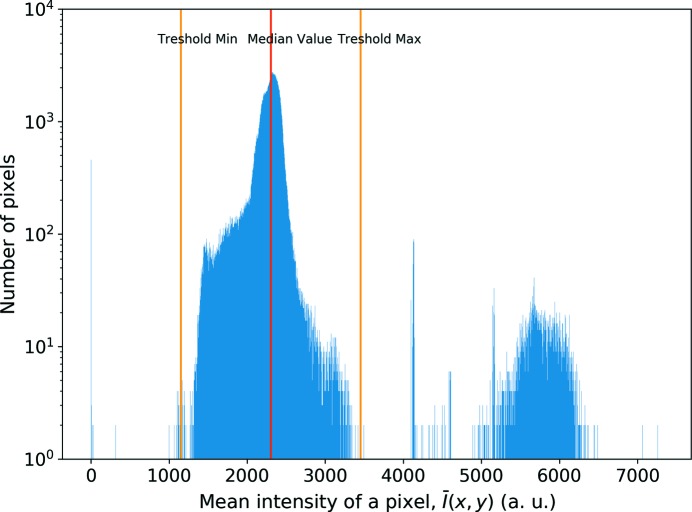
Histogram of mean pixel intensities for a set of 200 flat-field images. The second peak around 5500 intensity corresponds mostly to the ‘double pixels’.

**Criterion I** In order to determine such pixels we have made different measurements under different conditions. Firstly, an acquisition was made without any sample on the goniometer and the detector was put at a fixed position away from the direct incoming beam (*i.e.* recording an almost ‘flat’ scattering signal). Two hundred such flat-field images were recorded under these conditions with 60 s of exposure each. A mean value and a standard deviation were obtained for a given pixel of (*x*,*y*) coordinates based on these 200 images. A histogram of the mean intensity values 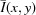 is shown in Fig. 6 ▸. Since the detector receives neither direct nor strongly diffracted beam, one expects the intensity levels to be rather uniform across the entire detector. However, because of the existence of the hot pixels, there are different intensity levels observed. Therefore we define a reference value (median value) for the intensity of a given pixel. Subsequently, the pixels above a certain threshold (1.5× the median value) are masked as hot pixels and the pixels lower than 0.5× the median value are masked as cold pixels. With this method, 12807 pixels to be masked are identified.

**Figure 7:**
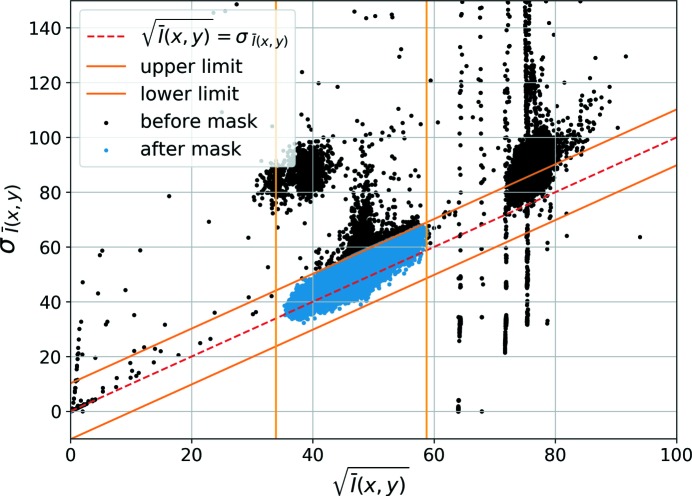
Mean intensity of a given pixel, 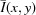, versus its standard error, 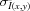 (the black points show all the pixels prior to masking and the blue pixels are those after applying the criteria I and II).

**Criterion II** Another analysis was made with the same set of 200 images to determine a noise model for the whole acquisition chain, from the X-ray sensitive surface of the detector up to the image storage in the computer. The counting of photons falling on a certain pixel shows a Poisson distribution with the standard error of measured intensities being equal to σ_*I*_ = *K*(*I*)^1/2^ (Petit *et al.*, 2015 ▸). For the recorded set of flat-field images, Fig. 7 ▸ shows the 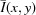 values versus the standard error on 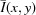, marked as 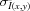. The 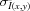 = 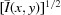 graph is also indicated on the same image. Since 95% of the data points are located near this line, we find *K* ≃ 1, which is the value expected for a single-photon-counting detector. Upper and lower limits obtained by ± a tolerance value are also plotted on the graph. The tolerance value is chosen to be 0.2× the median value of 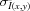 for this study. As a result, 13395 pixels outside these limits were found to violate the Poisson error model and therefore masked. Of these pixels, 3552 are unique, *i.e.* were not masked with the Criterion I.

**Criterion III** Similar to the flat-field study, dark-field images were recorded during which the shutter for the primary beam was kept closed. One hundred images were recorded for each exposure time of 1, 5, 10, 30 and 60 s totalling 500 images. Since there is basically no electronic noise in these types of detectors, counted intensity is either due to ambient noise (cosmic radiation, *etc*.) or due to pixel fault. Some 426 pixels with an intensity higher than 500 counts were considered faulty (usually hot pixels) and masked. It should be noted that all of these pixels are already masked by the two previous criteria.

**Figure 8:**
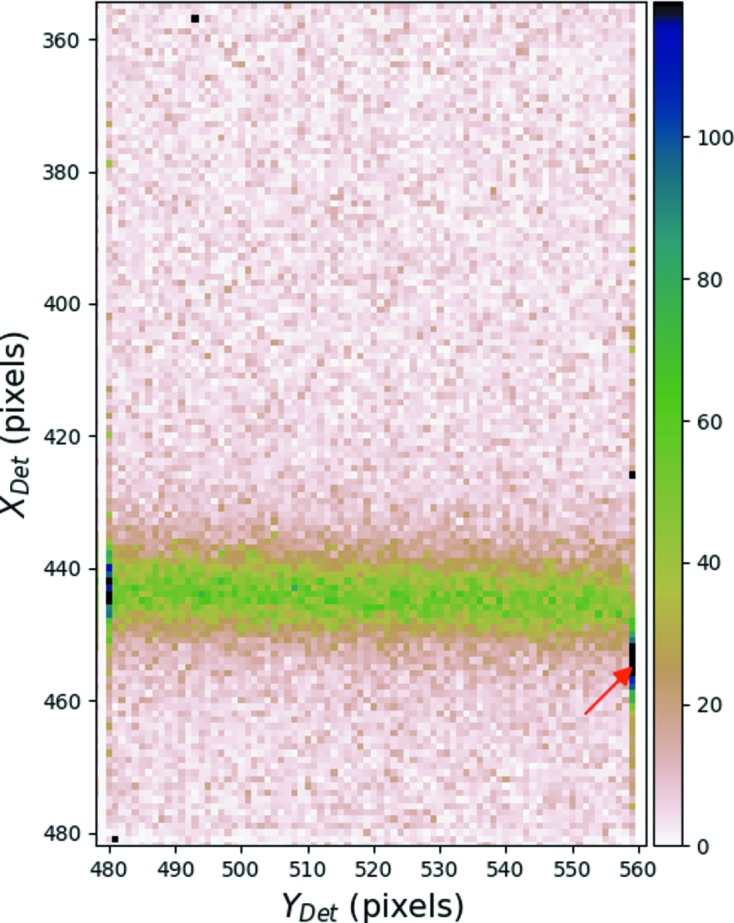
Zoom of the diffraction pattern shown in Fig. 5 ▸ in the vicinity of the last chip of a module.

**Criterion IV** In addition to quantitative criteria introduced above, a careful observation of numerous selected diffraction patterns was made. During these checks, an unusual behavior is observed. On the border of the last chip of a module there is a shift of a few pixels in recorded intensities along the *X* direction of the detector, as shown in Fig. 8 ▸. In this figure, the shift of intensity values in the last column of pixels is marked by a red arrow. This behavior is observed for the last chips of all the modules and is believed to be a flaw related either to a manufacturing error or to the reading of the data by the detector software. Thus the last column of pixels of *all* the chips of all modules are masked as a precaution.

The final toll of all masked pixels is 24040 out of a total of 537600. Therefore 4.47% of all the pixels are masked.

**Figure 9:**
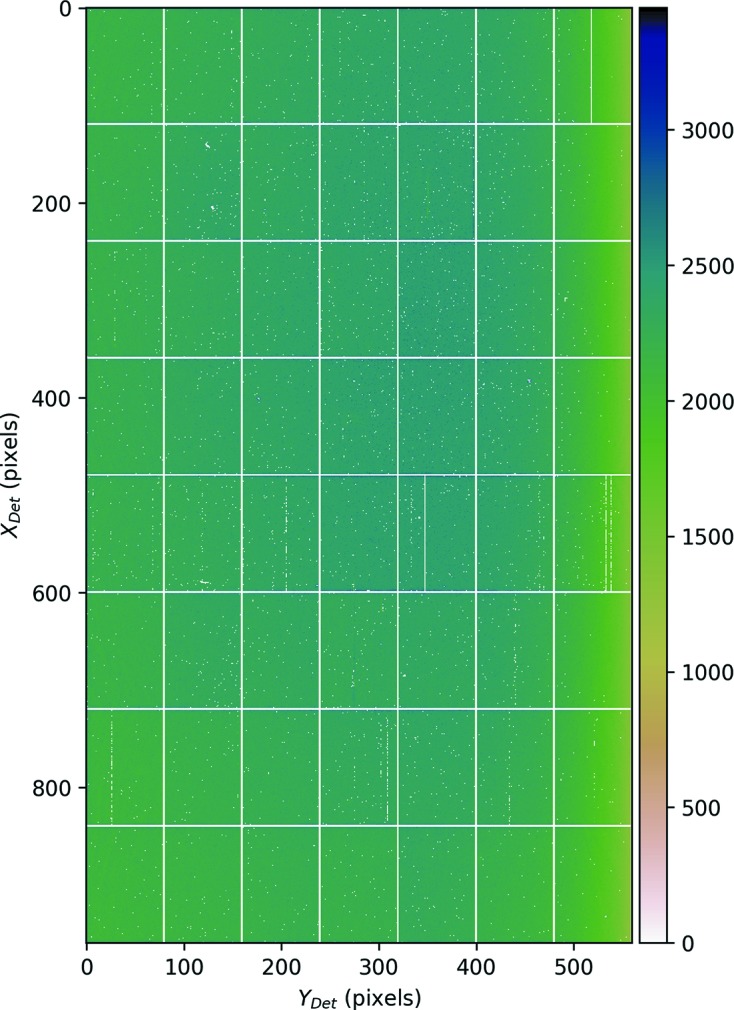
Average of 200 flat-field images after pixel masking is applied.

**Intensity normalization** Fig. 9 ▸ shows the distribution of the intensities for the mean of 200 flat-field images, after the mask is applied. According to this there is a clear gradient in intensity values, especially along the detector *y*-axis. This could be due to positioning of the detector relative to its surroundings, *e.g.* a part of the goniometer casting a shadow on the detector. In order to correct for this effect we have defined a pixel normalization factor, *N*(*x*, *y*), for each of the non-masked (active) pixels, by dividing the mean value of the active pixels of the average flat-field images by the mean value of that particular pixel,
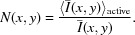
These normalization factors are multiplied by the intensity obtained from that particular pixel for all recorded patterns.

### 3.2. Geometrical adjustments   

After the masking and intensity normalization, Bragg diffraction angle, 2θ, and azimuthal angle, χ, need to be calculated. For this calculation the detector is positioned at zero angular position to receive the attenuated primary beam directly. The center of the peak caused by the direct beam was calculated by a 2D Gaussian fit and was accepted as the point where 2θ = 0 and χ = 0.

**Figure 10:**
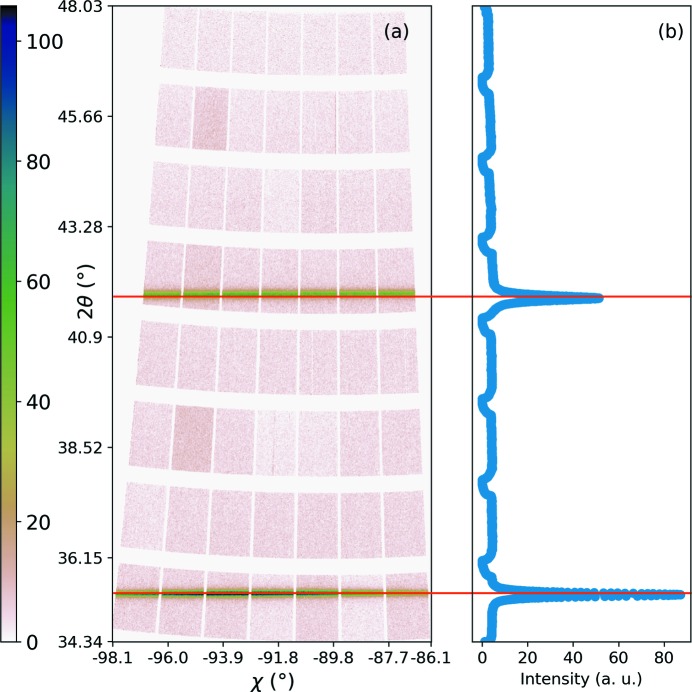
(*a*) 2D diffraction pattern and (*b*) 2θ [vertical axis, common scale with (*a*)] versus intensity plot after azimutal integration of the intensity (along χ).

Using this information, along with detector metrology (pixel size, module, gaps, double pixels) and experimental (sample-to-detector distance, wavelength of the beam, goniometer angles) parameters, 2θ and χ values for all the pixels were calculated. The software *pyFAI* (Ashiotis *et al.*, 2015 ▸) as well as a custom-made code was used for this purpose. The ‘Distortion’ class of the *pyFAI* package was used to account for the gaps and the double pixels in the detector, and the final 2θ and χ were calculated by the ‘AzimuthalIntegrator’ class. To show the 2D diffraction images regrouped along the 2θ and χ axes the ‘integrate2d’ function was used. Such an image is given in Fig. 10(*a*) ▸. In order to integrate along the azimuthal axis (χ) and obtain a conventional 1D diffraction pattern, the ‘integrate1d’ function is applied and the result is shown in Fig. 10(*b*) ▸. The drop in the background level is due to gaps between the modules where fewer pixels could record intensity from that particular 2θ.

### 3.3. Peak fitting   

According to equation (2) above one needs 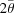 values in order to calculate 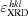. For the current study, experimentally measured peak profiles were fitted using a Pearson VII function, 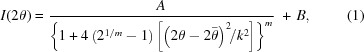
having a maximum intensity *A* + *B* and a FWHM of *k*, with *B* being the constant background level. Three different functions were tried (Gauss, Lorentz and Pearson VII) and Pearson VII produced the best results both at peak onsets and peak maxima. Moreover, the Pearson VII distribution becomes identical to the Lorentzian when the form parameter *m* = 1 and to the Gaussian for *m* → +∞ (practically *m* > 10).

**Figure 11:**
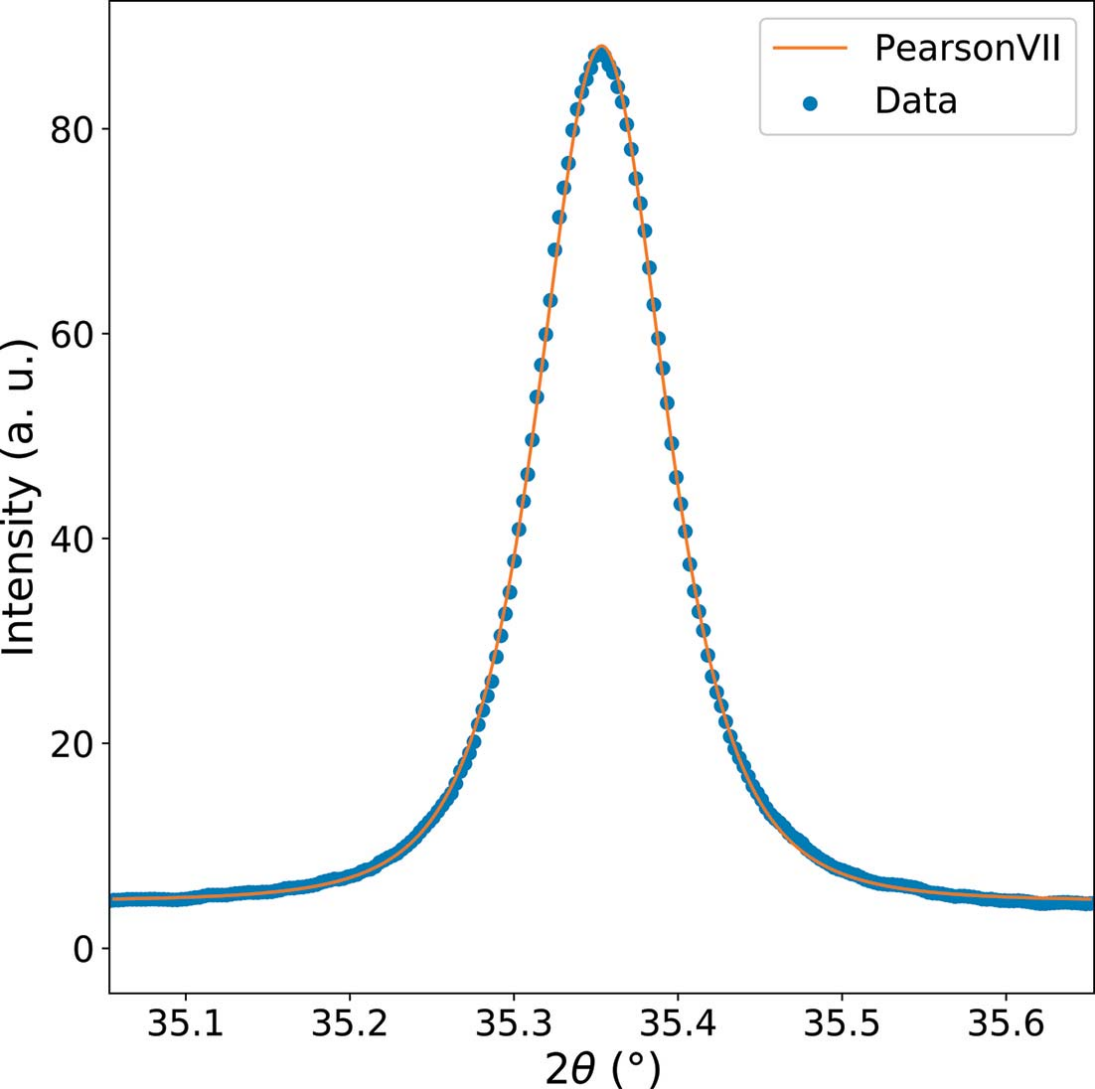
Peak fit on the Cu 220 reflection with the Pearson VII function.

Parameters *A*, *B*, *k* and *m* are fitted on experimental data by minimizing the objective function 
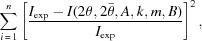
where *n* is the number of measurement points in the powder pattern. The 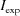 factor in the denominator represents the variance (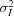) of the measured intensity data according to Poisson noise, as shown by the discussion of Criterion II in Section 3.1. A typical example of peak fitting is shown in Fig. 11 ▸, for a 220 peak. As shown in Appendix *A* [App appa], the errors in peak positions are ∼3 × 10^−4^ degrees corresponding to errors of ∼6 × 10^−6^ in terms of deformation. The data obtained for the (311) planes give similar results.

### 3.4. Micromechanical interpretation of diffraction data   

One of the objectives of this study is the estimation of the stress applied to the specimen during the ultrasonic fatigue test. To obtain this information from the shift of diffraction peaks, one needs to rely on a scale transition model (*e.g.* Letouzé *et al.*, 2002 ▸; Faurie *et al.*, 2009 ▸). Within the material, the local stress field (*i.e.* at a fine intra-granular scale) is heterogeneous due to many factors, one of them being the elastic anisotropy at the grain scale. Consequently, lattice spacings *d* ^*hkl*^ are also non-uniform and distributed according to the stress heterogeneity within individual grains or within grains sharing the same crystallographic orientation. The grains at the origin of the measured {*hkl*} diffraction peak, constituting the diffracting volume denoted hereafter Ω, are those for which the normal of an (*hkl*) plane lies parallel to the diffraction vector **K** = **k**_d_ − **k**_i_, with **k**_d_ and **k**_i_ being the diffracted and incident wavevectors of norm 1/λ, respectively. The shift 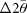 of a diffraction peak during the mechanical test, or more precisely the shift of its centroid 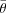 (Le Bourlot, 2012 ▸), exactly provides a measurement of the shift of the *mean* lattice spacing 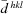 within the diffracting volume,
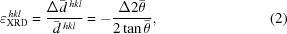
with 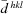 = 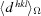, where 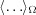 denotes the volume average over grains belonging to Ω which itself is a function of the reflection therefore Ω(*hkl*). The so-called *lattice strain* 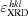 is a projection along **K** of the mean strain tensor 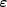 over Ω, *i.e.* 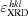 = 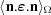, with **n** being a unit vector parallel to **K**. Next, one has to introduce the (fourth-order) stress concentration tensor **B** which links the local stress tensor 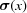 at position *x* within the material with the macroscopic (or applied) stress 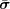, 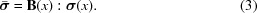
Combining the above equations, one obtains
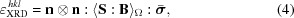
with **S** the elastic compliance tensor of grains, ⊗ the dyadic product and : the twice-contracted product. The term 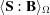 is usually called the X-ray elastic constant (XEC) in the literature; it requires an evaluation of the mechanical interaction between the grains for **B**. In the present study, this is achieved with the self-consistent model which performs very well for polycrystalline aggregates (Lebensohn *et al.*, 2005 ▸, 2011 ▸; Brenner *et al.*, 2009 ▸). Assuming uniaxial loading conditions for the sample and considering that the diffraction vector is very close to the surface normal, we can simplify the stress calculation from 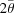 measurement to
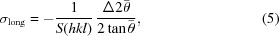
where 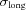 is the stress along the longitudinal axis of the sample and *S*(*hkl*) is the XEC for the *hkl* direction in question. The self-consistent model yields values *S*(311) = −2.55 × 10^−6^ MPa^−1^ and *S*(220) = −2.74 × 10^−6^ MPa^−1^ for the 311 and 220 reflections, respectively.

## 4. Results and discussion   

**Figure 12:**
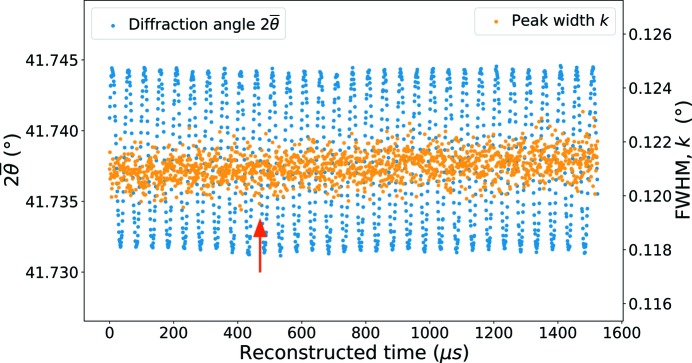
Reconstructed cycles showing the effect of fatigue loading in terms of 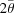 (blue) and peak width (*k*, orange) versus reconstructed time for the (311) plane and a displacement amplitude of 3.5 µm. The red arrow points out the beginning of a particular cycle (*t* = 470 s) that is used in the following images and discussion (see text for details).

As mentioned in the previous sections, the developed experimental device and the analysis enable us to reconstruct the evolution of the diffraction patterns during one cycle and thus to follow the evolution of two diffraction peaks and quantify the evolution of their position and their FWHM (denoted by 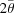 and *k*, respectively). Fig. 12 ▸ shows their evolution *versus * time in the case of an ultrasonic fatigue test with an imposed displacement amplitude of 3.5 µm. The abscissa of the plot is denoted ‘Reconstructed time’ and is defined as the time for a given position in the cycle. Fig. 12 ▸ exhibits a sinusoidal evolution of 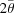 according to the reconstructed time at a frequency very close to the applied loading frequency. This sinusoidal fluctuation occurs around a constant value, noted 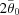, of about 41.7381°. The entire experiment shown in Fig. 12 ▸ contains 30 reconstructed fatigue cycles. During these 30 cycles, the mean value of the 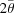 amplitude is about 0.0063° with a standard deviation of 0.0004°. As shown in Section 3.4, this evolution of 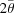 can be directly related to the lattice elastic strain and the longitudinal normal stress.

Another interesting parameter obtained by Pearson VII fits is the FWHM (denoted *k*). As shown in the graph of Fig. 12 ▸, the peak broadening does not show a very strong cyclic variation but it can be observed that this value evolves quasi-linearly with time. This increase could be explained by two factors: distribution of the elastic strain and the increase in plastic deformation in the sample (accumulation of defects, *etc*.). From Fig. 12 ▸, it is possible to estimate an increase of the *k* parameter of 0.00055° during the 30 reconstructed cycles. The standard deviation of this parameter is about 0.00048° which is close to its increase. In the case of the test presented in Fig. 12 ▸, the slope of the *k* parameter is about 0.358° s^−1^ in the reconstructed time scale and thus 4.1 × 10^−12^ degrees (or ∼2 × 10^−14^ strain) per cycle really applied to the specimen.

**Figure 13:**
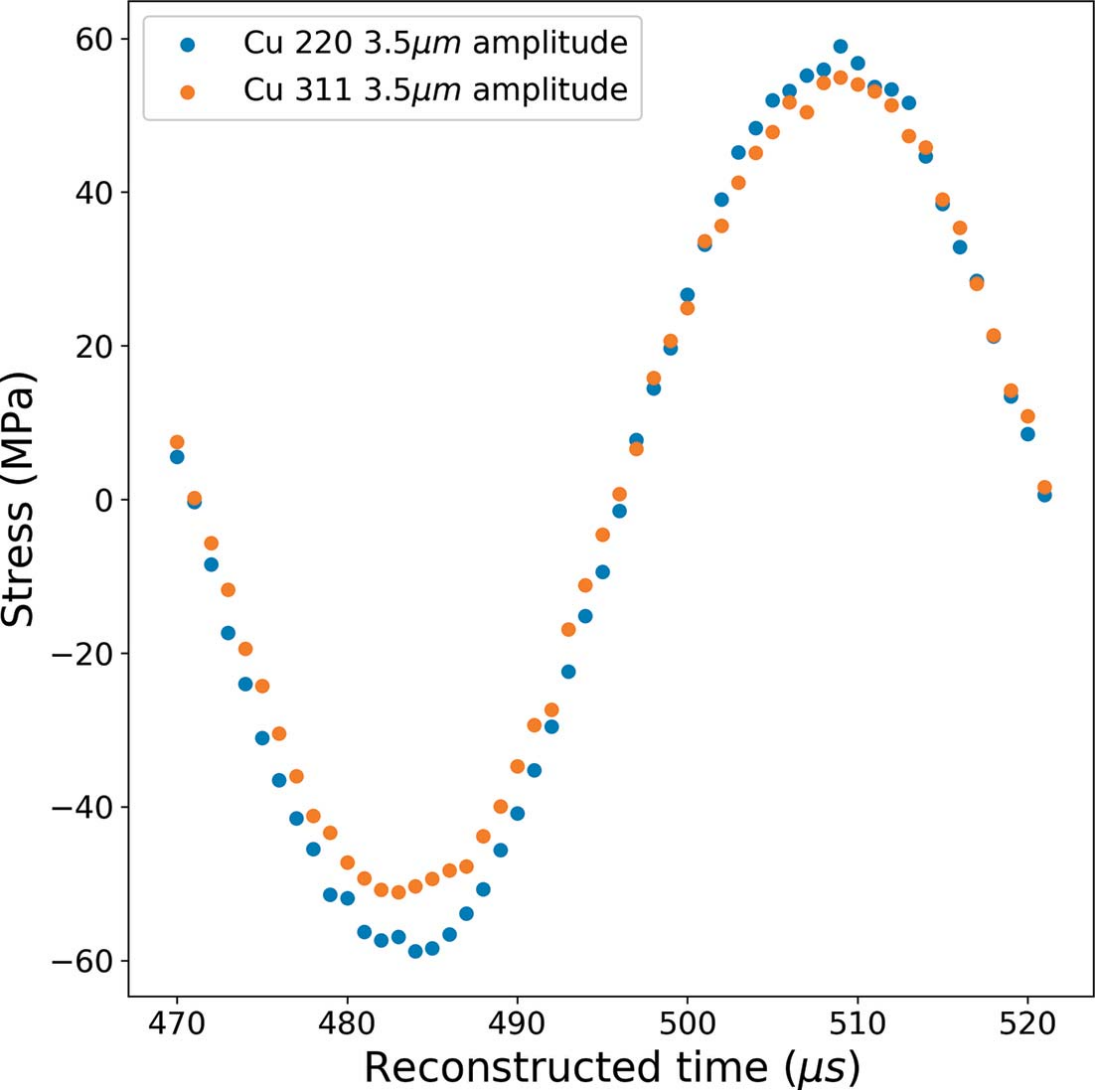
Longitudinal stress during an isolated cycle versus reconstructed time for two reflections (220 and 311).

After having estimated the 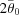 value, we can calculate the lattice elastic strain 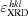 by the help of equations (2) and (5) and plot the temporal evolution of longitudinal normal stress (σ_long_) to visualize the effect of cyclic loading on the crystal structure. Fig. 13 ▸ represents reconstructed loading cycles for both of the reflections (311 and 220) for one isolated cycle shown in Fig. 12 ▸ (the considered cycle is identified in Fig. 12 ▸ by a red arrow). The amplitude of the applied displacement on the edge of the specimen is about 3.6 µm. Fig. 13 ▸ shows that the estimations of the stress from the two Bragg peaks are very similar. For the (220) and the (311) planes the stress amplitudes are 58.9 MPa and 53.0 MPa, respectively, which corresponds to a relative error of about 10%. This error can most probably be explained by the weak crystallographic texture in the specimen that is not taken into account in the calculation of the XEC in this illustrative example.

**Figure 14:**
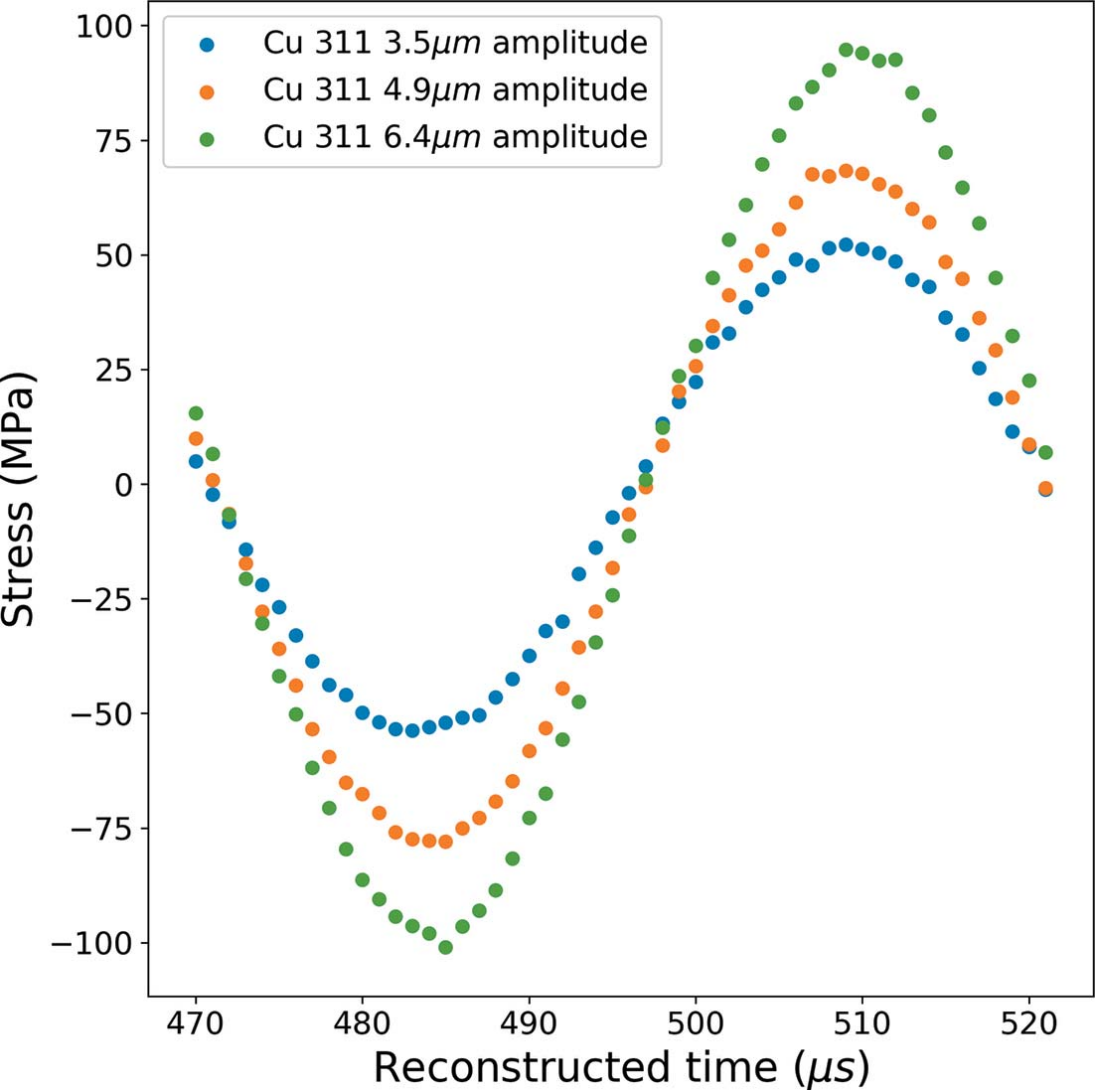
Longitudinal stress versus reconstructed time curve for different imposed displacements [for the (311) planes] for a single cycle.

Different amplitudes of displacement were also applied to different samples by the ultrasonic fatigue machine, namely 3.5 µm, 4.9 µm and 6.4 µm. The obtained results during one reconstructed cycle are shown in Fig. 14 ▸ and illustrate clearly the increase of the longitudinal normal stress by increasing the vibration amplitude.

**Figure 15:**
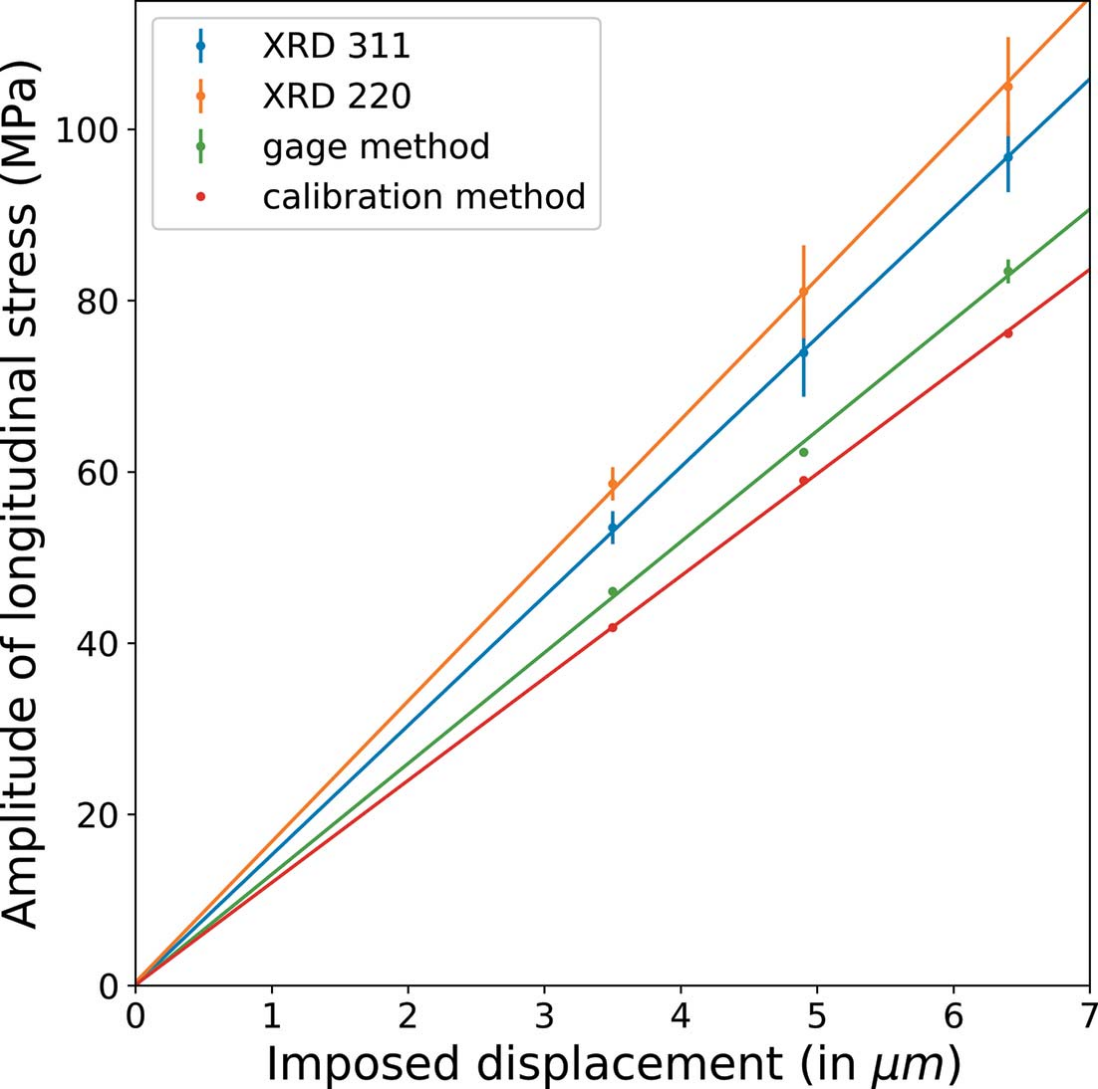
Amplitudes of 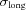 measured by the XRD method (for both 220 and 311 reflections), gage method and calibration method plotted with respect to displacements imposed by the ultrasonic fatigue machine.

To highlight again this effect, Fig. 15 ▸ represents the evolution of the amplitude of the stress with the applied displacement amplitude. In this figure, the longitudinal normal stress amplitudes are estimated for 30, 25 and 80 reconstructed cycles for displacement amplitudes of 3.5 µm, 4.9 µm and 6.4 µm, respectively. The error bars give the dispersion on the stress amplitude during all the reconstructed cycles. Moreover, the longitudinal stress calculated from the strain gage signal considering an elastic behavior with a Young modulus *E* = 120 GPa are also added in Fig. 15 ▸ as well as a calculation from the displacement with a harmonic elastic model. The results highlight a very good linearity of the measured amplitudes with respect to the imposed displacement. This linearity is related to the linearity of the response of the ultrasonic fatigue machine and the quasi linearity of the mechanical behavior of the material. However, a difference of 20–25% between the conventional methods (gage and calibration) and the XRD method is visible on the graph. This factor between XRD- and gage-measured stress is constant for a given reflection over all amplitudes and is likely due to micro-structural reasons that lead to errors in the estimation of XECs. While estimating the XECs by the self-consistent model, certain assumptions are made on the micro-structure. These conditions may not be accurate for the material analyzed due to texture and grain size distribution. Better estimation of XECs through static loading measurements and/or by using different micro-mechanical models which take into account the microstrucural texture of the material could be possible in future.

## 5. Conclusion   

Using the proposed method of time-resolved XRD, we show that it is possible to obtain diffraction patterns with a temporal resolution of ∼1 µs during an ultrasonic fatigue test where the loading frequency is about 20 kHz. After geometrical correction and bad pixel detection, the XRD image enables the displacement of diffraction peaks and broadening to be determineed as a function of time. The diffraction peak shift gives information on the lattice strain with a resolution better than 10^−5^. Moreover, using a micro-mechanical homogenization technique, the longitudinal normal stress in the center of the fatigue specimen can be estimated.

The longitudinal normal stress measured by the XRD method versus the imposed displacement graphs shows very good linearity confirming the stability of the method at different levels of loading. The stress values measured by other methods (strain gage and displacement) are consistently lower by a factor of 20–25%. This factor between XRD- and gage-measured stress is constant for a given reflection over all amplitudes and is likely due to the difficult estimation of XECs.

## Figures and Tables

**Table 1:** Errors and uncertainties on two parameter after peak fitting on experimental and simulated diffraction patterns of a Cu sample obtained with different exposure times

	Total exposure time (s)			
	0.02	0.004	0.001	0.0002
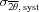 (°)	2.80 × 10^−4^	5.32 × 10^−4^	1.13 × 10^−3^	2.91 × 10^−3^
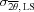 (°)	1.44 × 10^−4^	3.21 × 10^−4^	6.30 × 10^−4^	1.38 × 10^−3^
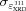	6.32 × 10^−6^	1.20 × 10^−5^	2.56 × 10^−5^	6.57 × 10^−5^

## Appendix A Effect of intensity measurement uncertainties on peak position measurement

In order to determine optimal parameters for our experiment (in terms of signal statistics, signal-to-noise ratio, duration of the experiment to access VHCF, *etc*.) we have recorded diffraction patterns with different exposure times (0.0002, 0.001, 0.004 and 0.02 s) on a polycrystalline Cu sample without ultrasonic vibration, therefore under a static state of deformation. The measured diffraction patterns were treated and analyzed as described in Section 3. Pearson VII functions were used to fit the measured diffraction peaks. Least-squares (LS) fitting uncertainties obtained for the peak position parameter () for the 311 reflection are given in Table 1 ▸ as . According to this, the precision on the determination of the angular position of the diffraction peak () is improved up to ten times between 0.0002 and 0.02 s of exposure. These uncertainties are characterized by the errors occurring due to random procedures (counting statistics, *etc*.) and LS fitting parameters (choice of the fitting function, initial parameters, stability of the fit, *etc*.). They refer to the precision of the experiment.

To characterize the effect of the random noise in our diffraction patterns on the accuracy of our scattering angle measurement we need an initial 2θ value. Therefore a modeling approach is used. An ideal 2D diffraction pattern is created for the same 311 reflection with the initial value 2θ_init_ = 41.65° using the Pearson VII function and the experimental geometrical parameters for the goniometer. Random counting errors are added to the intensity values according to the Poisson model taking into account the simulated exposure time. After adding the noise the 2D pattern is treated in the same way as the experimental patterns, using the same geometrical parameters for the goniometer. The fitted value after treatment, , is then compared with the initially set value to estimate the systematic error due to noise  = .

The larger of the these two types of errors ( and ) is propagated to strain calculations using equation (2). The results are also given in Table 1 ▸. According to this, the final errors on strain for 0.02 s of total exposure is 6.32 × 10^−6^. The results are similar for the 220 reflection.

According to these results, 0.02 s of exposure time is chosen for each recorded image. Since we are counting with a detector aperture of 10^−6^ s cycle^−1^, the 0.02 s photon counting time corresponds to 0.02 s/10^−6^ s cycle^−1^ = 2 × 10^5^ cycles applied by the machine during the recording of one diffraction image. Since we are at 20 kHz, then 2 × 10^5^ cycles/2 × 10^5^ cycles s^−1^ = 1 s of total pattern recording time when the time is needed.
